# BCAS2 Regulates Delta-Notch Signaling Activity through *Delta* Pre-mRNA Splicing in *Drosophila* Wing Development

**DOI:** 10.1371/journal.pone.0130706

**Published:** 2015-06-19

**Authors:** Meng-Hsuan Chou, Yi-Chen Hsieh, Chu-Wei Huang, Po-Han Chen, Shih-Peng Chan, Yeou-Ping Tsao, Hsiu-Hsiang Lee, June-Tai Wu, Show-Li Chen

**Affiliations:** 1 Graduate Institute of Microbiology, College of Medicine, National Taiwan University, Taipei, 100, Taiwan; 2 Department of Ophthalmology, Mackay Memorial Hospital, Taipei, 104, Taiwan; 3 Institute of Molecular Medicine, College of Medicine, National Taiwan University, Taipei, 100, Taiwan; 4 Department of Medical Research, National Taiwan University Hospital, Taipei, 100, Taiwan; National Institutes of Health (NIH), UNITED STATES

## Abstract

Previously, we showed that BCAS2 is essential for *Drosophila* viability and functions in pre-mRNA splicing. In this study, we provide strong evidence that BCAS2 regulates the activity of Delta-Notch signaling via *Delta* pre-mRNA splicing. Depletion of *dBCAS2* reduces *Delta* mRNA expression and leads to accumulation of *Delta* pre-mRNA, resulting in diminished transcriptions of Delta-Notch signaling target genes, such as *cut* and *E(spl)m8*. Furthermore, ectopic expression of human *BCAS2* (*hBCAS2*) and *Drosophila BCAS2* (*dBCAS2*) in a *dBCAS2*-deprived fly can rescue *dBCAS2* depletion-induced wing damage to the normal phenotypes. These rescued phenotypes are correlated with the restoration of *Delta* pre-mRNA splicing, which affects Delta-Notch signaling activity. Additionally, overexpression of *Delta* can rescue the wing deformation by deprivation of *dBCAS2*; and the depletion of *dBCAS2* can restore the aberrant eye associated with *Delta*-overexpressing retinas; providing supporting evidence for the regulation of Delta-Notch signaling by dBCAS2. Taken together, dBCAS2 participates in *Delta* pre-mRNA splicing that affects the regulation of Delta-Notch signaling in *Drosophila* wing development.

## Introduction

The product of breast carcinoma amplified sequence 2 (BCAS2), a 26 kD small nuclear protein, was initially identified as a transcriptional co-activator involved in the transcriptional regulation of the estrogen receptor (ER) [1]. The increased BCAS2 expression is correlated with the aggressive breast cancer cells [2]; and prostate cancer [3]. Recently, we showed that BCAS2 interacts directly with the tumor suppressor p53, regulating its stability and transcriptional activity. Depletion of BCAS2 induces apoptosis in p53 wild-type cancer cell lines (such as MCF7 and A549), but leads to G2/M growth arrest in p53-null H1299 cells and p53 mutant C33A cells. This evidence indicates that beyond p53, BCAS2 may be involved in other mechanisms of cell growth regulations.

BCAS2 is also named as SPF27 that was potentially associated with splicing [4]. The spliceosomal core complex including Prp19 (PSO4, human orthlog), Cef1 (CDC5L), Prp46 (PLRG1), and Snt309 (BCAS2), contributes RNA splicing in yeast and human cells [5–17]. The yeast Prp19p-associated complex forms the stable complexes with the U5 and U6 snRNP, after dissociating from U4 snRNP during RNA splicing process [7, 8]. Hence, interference of the yeast BCAS2 ortholog (*Cwf7* and *Snt309)* using genetic methods can cause the accumulation of pre-mRNA [9, 10, 15, 17]. Recently, we showed that *Drosophila* BCAS2 (*CG4980*, human BCAS2 ortholog) is essential for the viability of *Drosophila melanogaster*. The ubiquitous depletion of *dBCAS2* in *Drosophila* leads to larval lethality, and the deprivation of *dBCAS2* in wing formation causes wing deformities which are correlated with impaired pre-mRNA splicing. More importantly, overexpression of human *BCAS2* rescues these defects, indicating that *Drosophila* and human BCAS2 share a similar function in RNA splicing, which affects cell viability [11].

Recent evidence shows that the Prp19 core complex may be involved in the developmental process. For example, hPrp19 (PSO4)- and PLRG1-knockout mice suffer early embryonic lethality at the blastocyst stage [12]. PLRG1 also is an essential regulator of cell proliferation, p53-dependent apoptosis during embryonic development and adult tissue homeostasis [14]. Moreover, CDC5L also takes part in the regulation of G2/M progression [6]. Our recent study showed that BCAS2 is a member of the hPrp19 core complex and depletion of *dBCAS2* in *Drosophila* results in larval lethality and deformity of adult wings [11]. All the above studies suggest that the core members of the hPrp19 complex are important for the developmental process. However, the effect of hPrp19-mediated pre-mRNA splicing on development remains unknown. Current report demonstrated that BCAS2 gene linkage region (1p13.2) is associated with autism [18]. It is very interesting to investigate the role of BCAS2 in development process.

Delta-Notch signaling is an evolutionarily conserved developmental signaling pathway, and is involved in cell fate determination and patterning interaction in *Drosophila* [19]. The short-range communication between cells by Delta-Notch signaling begins with the binding of Delta ligand from one cell to the Notch receptor on the membrane of an adjacent cell. This binding can activate a series of proteolytic enzymes to cleave Notch and generate the Notch intracellular domain (NICD), which then travels into the nucleus to trigger the expression of the target genes, such as *Enhancer of split m8* ([*E(spl)*]bHLH) and *cut* (a homeobox-containing transcription factor) [20]. Therefore, in this study, we sought to explore how BCAS2 regulates *Delta* pre-mRNA splicing, resulting in the regulation of Delta-Notch signaling during *Drosophila* wing development.

## Results

### BCAS2 is involved in the regulation of Delta-Notch signaling

**Fig 1:**
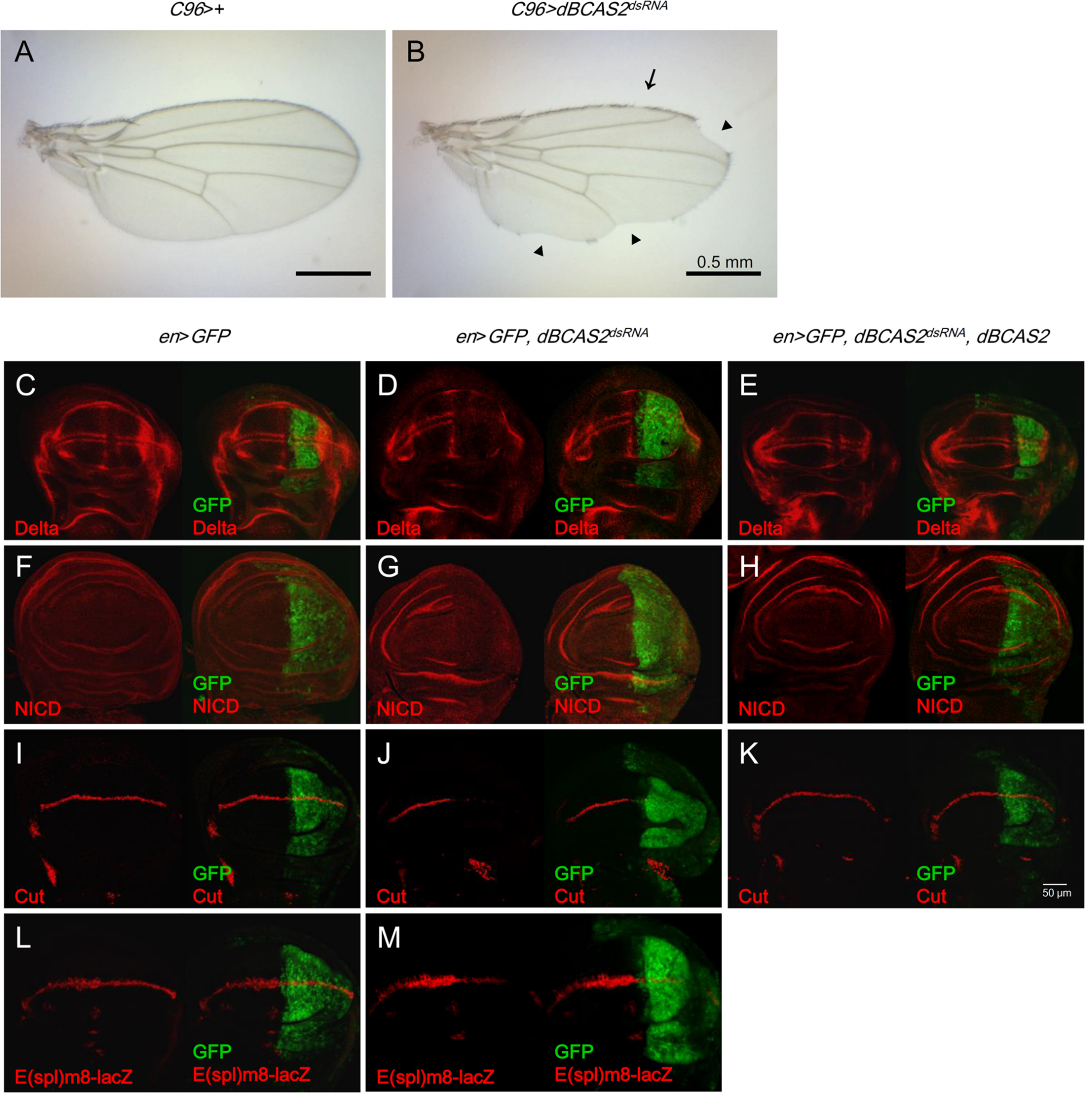
BCAS2 is involved in the regulation of Delta-Notch signaling. (A, B) Depletion of *dBCAS2*, driven by the wing margin *C96-GAL4* driver, generates a phenotype similar to reduction of Delta-Notch signaling activity. (A) Control wing (*C96*>+). (B) *dBCAS2*-depleted wing (*C96*>*dBCAS2* ^*dsRNA*^). Arrow, loss of margin bristles; arrow head, wing notching. Scale bar, 0.5 mm. (C to M) Depletion of dBCAS2 decreases the activity of Delta-Notch signaling that can be restored by dBCAS2. The late third instar larval wing discs were driven under the control of *engrailed-GAL4* and immunostained with the indicated antibodies. (C, F, I, L) Control (*en*>*GFP*); (D, G, J, M) *dBCAS2*-depletion (*en*>*GFP*, *dBCAS2* ^*dsRNA*^); (E, H, K) Coexpression of *dBCAS2* with *dBCAS2* ^*dsRNA*^ (*en*>*GFP*, *dBCAS2* ^*dsRNA*^, *dBCAS2*). (C, D, E) Anti-Delta antibody. The normal pattern of Delta (red) can be seen in the cells of presumptive veins and on either side of the D/V boundary; (F, G, H) Anti-NICD antibody. The Notch expression can be observed in the presumptive intervein territories; (I, J, K) Anti-Cut antibody. The Cut expression is located in the stripe of D/V boundary; and (L, M) E(spl)m8-lacZ. *LacZ* reporter of *E(splm8)*, a Delta-Notch signaling target gene is expressed along the D/V boundary. Left of the panel: red stain with the indicated antigen expression in the anterior and posterior compartments. Right of the panel: the merged images reveal the simultaneous expression of the protein of interest (red) and GFP fluorescence (green) in the posterior. Images were taken by confocal microscopy, scale bar, 50 μm.

BCAS2 is essential for *Drosophila* viability because depletion of *dBCAS2* in the entire body, driven by *Act5C-GAL4*, leads to no adult fly survival [11]. We speculated that BCAS2 might play a role in the development process and explored the signaling regulated by BCAS2. Interestingly, we found that when *dBCAS2* was depleted in the region of the wing imaginal disc by the *C96-GAL4* driver [21], which affects the dorsal-ventral wing margin, the adult wing in *C96>dBCAS2* ^*dsRNA*^ showed loss of margin bristles (Fig 1B arrow) and wing notching (Fig 1B arrow head). These phenotypes are similar to those caused by a reduction of Delta-Notch signaling activity, as reported previously [22–24]. Therefore, we examined the expression of genes involved in Delta-Notch signaling when dBCAS2 expression was reduced in *Drosophila* model systems. We chose *engrailed-GAL4* (*en-GAL4*) to drive the expression of *dBCAS2* ^*dsRNA*^ in the posterior compartment of the wing disc in third instar larvae [25]. The results were that normal expression patterns of Delta and Notch in presumptive vein and intervein territories, respectively, in the anterior compartment of late-third instar wing discs (Fig 1C and 1F) were consistent with a previous report [26]. But when *dBCAS2* was depleted in the posterior compartment, the proteins level of Delta (Fig 1D) and NICD (Fig 1G) were decreased. Taken together, dBCAS2 may regulate Delta-Notch signaling.

To corroborate dBCAS2-regulation of Delta-Notch signaling further, we measured the expression of Cut in a stripe along the dorsal/ventral (D/V) boundary in the wing disc [27]. When dBCAS2 expression was reduced under the control of *en-GAL4*, the expression level of Cut decreased (Fig 1J), indicating that the reduced expression of Delta and Notch by *dBCAS2* affects the expression of down-stream target genes. To confirm that dBCAS2 regulates Delta-Notch signaling activity, we examined a *LacZ* reporter of *E(splm8)*, another Delta-Notch signaling target gene [28], expression of which is along the D/V boundary (Fig 1L). The expression of *E(spl)m8-lacZ* decreased in the posterior compartment of which *dBCAS2* was down regulated (Fig 1M). Taken together, disruption of Delta-Notch signaling may be one of the reasons for the wing deformation mediated by depletion of *dBCAS2*.

### *Drosophila* and human BCAS2 rescues wing deformation induced by depletion of *dBCAS2* via Delta-Notch signaling

Previously, we showed that *hBCAS2* and *dBCAS2* have sequence similarity and hBCAS2 plays a similar role as dBCAS2 in the fly. hBCAS2 can rescue the wing deformation induced by depletion of *dBCAS2*. In this study, we generated a *dBCAS2* transgenic fly. When the expression of *dBCAS2* was driven by *ms1096-GAL4*, which is expressed in the pouch region of wing discs [29], the morphology of adult wings did not change (S1Aa and S1Ab Fig), and dBCAS2 protein expression was confirmed in the protein extracted from the third instar larvae of *dBCAS2* transgenic flies (*en*>*GFP*, *dBCAS2*) (S1B Fig). Hence, *dBCAS2* transgenic fly, like *hBCAS2*, presents the normal wing phenotypes. To determine whether dBCAS2 could rescue *dBCAS2* ^*dsRNA*^-induced wing deformation, *dBCAS2* ^*dsRNA*^ and *dBCAS2* were simultaneously expressed under the control of *ms1096-GAL4* to monitor the morphology of adult wings. Similar to the rescue effects of *hBCAS2* on *dBCAS2* ^*dsRNA*^ expressing flies, dBCAS2 could restore the wing deformation in flies expressing *dBCAS2* ^*dsRNA*^ (S1Cc Fig) and the restored wing phenotypes resembled the control (S1Ca Fig).

To confirm that dBCAS2 rescues the wing deformation via Delta-Notch signaling, the *dBCAS2* ^*dsRNA*^ and *dBCAS2* were co-expressed in the posterior compartment of the wing disc under the control of the *engrailed* promoter, as mentioned above. The results were that the levels of Delta, NICD and Cut were rescued in *en*>*dBCAS2* ^*dsRNA*^, *dBCAS2* flies (Fig 1E, 1H and 1K), compared to those expressed in *dBCAS2*-depleted wing discs (Fig 1D, 1G and 1J). Additionally, the transgenic flies of *hBCAS2* [11] and *dBCAS2* (S1Ab Fig) showed the normal wing morphology. The expression level of Delta, NICD and Cut in either *hBCAS2* (S2 Fig) or *dBCAS2* transgenic fly (data not shown) was the same as the control fly. Similar to the rescue effects of *dBCAS2* on *dBCAS2* ^*dsRNA*^ expressing flies (Fig 1E, 1H and 1K), *hBCAS2* also restored the expression levels of Delta, NICD and Cut from *dBCAS2* ^*dsRNA*^ (S3C, S3F and S3I Fig). Due to dBCAS2 or hBCAS2 transgenic fly can restore the declination of *Delta* gene expression and wing deformation caused by *dBCAS2* ^*dsRNA*^ fly. To explain why *dBCAS2* ^*dsRNA*^ construct does not interfere with UAS-BCAS2, it may be the reason that high amount BCAS2 expression from ectopic expressing dBCAS2 or hBCAS2 can compromise the reducing BCAS2 expression by RNAi effect. In summary, *dBCAS2* ^*dsRNA*^–induced wing deformation could be due to the reduced Delta-Notch signaling activity.

### BCAS2 regulates the expression of Delta through pre-mRNA splicing

**Fig 2:**
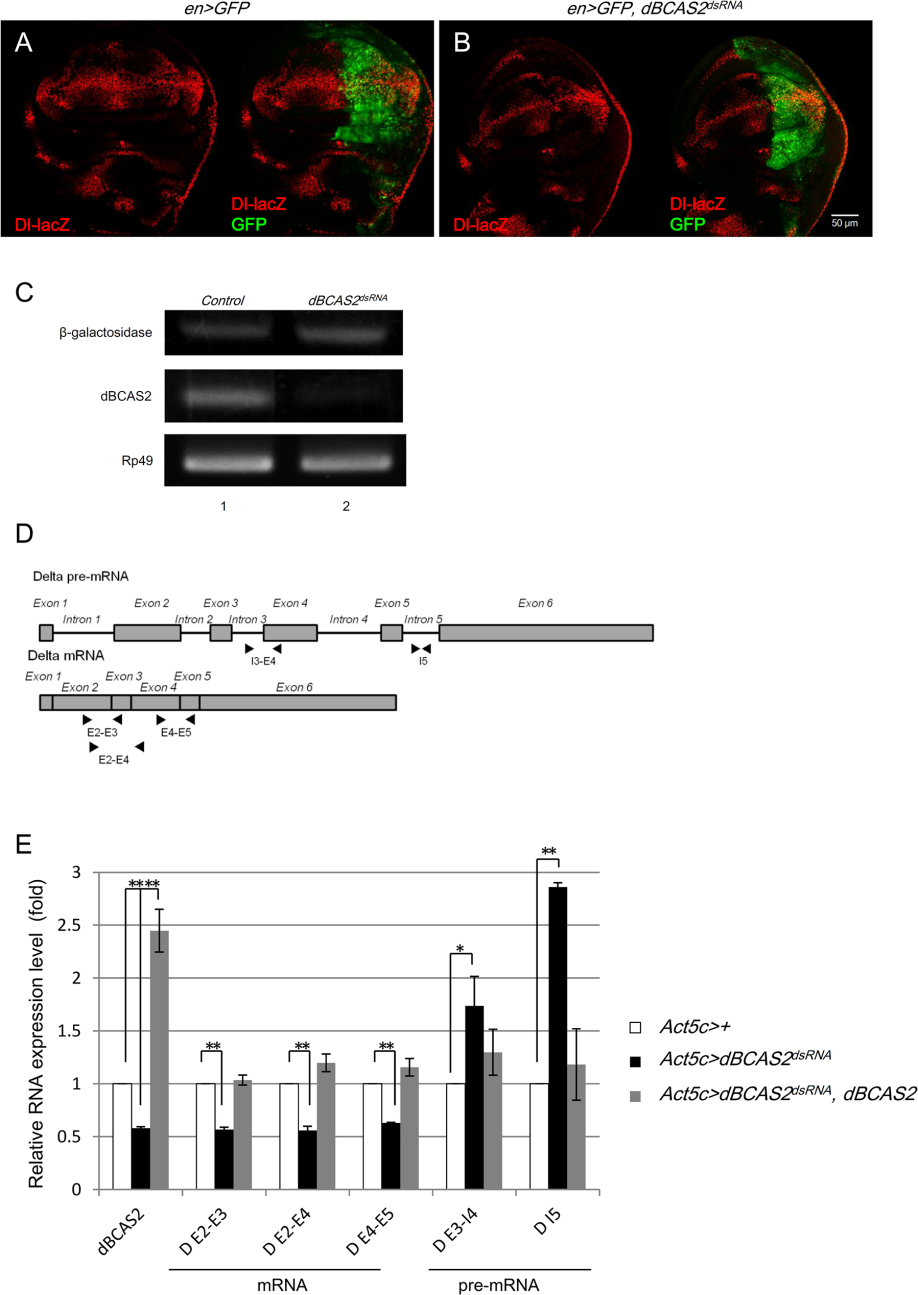
BCAS2 does not regulate the transcription initiation of *Delta* but is involved in *Delta* pre-mRNA splicing. (A) Control of *Dl-lacZ* (red) in the *en*>*GFP* wing disc stained with anti-β-gal antibody. (B) *en*>*GFP*, *dBCAS2* ^*dsRNA*^. In the *dBCAS2*-depleted posterior compartment, marked by GFP, the expression of *Dl-lacZ* (red) gives a signal of similar strength as the normal anterior compartment. The expression of GFP and β-galactosidase were merged and displayed in the right panel. Images were taken by confocal microscopy, scale bar, 50 μm. (C). RNA expression of β-galactosidase. RNAs were extracted from wing discs of third instar larvae and subjected to RT-PCR to confirm the RNA expression of β-galactosidase driven by *Dl* promoter in *Act>dBCAS2* ^*dsRNA*^ (lane 2) compared with the control (lane 1). The internal control, *rp49*. (D) Schematic diagram of primer design for detecting the intron-containing precursor mRNA (upper) and mRNA of *Delta* (lower). Primers, exons and introns are denoted with arrowheads, boxes and lines, respectively. (E) Coexpression of *dBCAS2* and *dBCAS2* ^*dsRNA*^ in larvae can rescue the phenotypes of mRNA decrease and pre-mRNA accumulation caused by *dBCAS2* ^*dsRNA*^. The pre-mRNA and mRNA of *Delta* were analyzed by quantitative RT-PCR and described in the Materials and Methods. Each genotype was under the control of *Act5c-GAL4* driver. White bar: *Act5c>+;* black bar: *Act5c>dBCAS2* ^*dsRNA*^; gray bar: *Act5c>dBCAS2* ^*dsRNA*^, *dBCAS2*. Data are shown as means and SD relative to the controls from three independent experiments. The P-values was measured by the Student’s t-test. *p<0.05, **p<0.01.

BCAS2 is a core component of the hPrp19/CDC5L splicing complex and functions in pre-mRNA splicing. Accordingly, dBCAS2 might be speculated to regulate the expression of *Delta* through the transcriptional process in the nucleus. Gene transcription could be modulated in two ways, promoter regulation and RNA splicing. The *Delta* gene transcription initiation assay was examined using *Delta-lacZ* as a reporter gene to determine whether the initiation of *Delta* transcription was impeded by the deprivation of dBCAS2. The expression level of β-galactosidase located at the stripe of the dorsal-ventral boundary in the dBCAS2-depleted fly (*en*>*GFP*, *dBCAS2* ^*dsRNA*^) (Fig 2B) was the same as control (Fig 2A). Moreover, the β-galactosidase expression did not differ between the anterior and posterior compartments of the wing discs of the *dBCAS2*-depleted fly (Fig 2B). To rule out a consequence of β-galactosidase protein stability rather than the continued transcription, we conducted β-galactosidase RNA by RT-PCR. As shown in Fig 2C, RNA expression level of the control and *dBCAS2*-depleted fly was the same. Thus, dBCAS2 regulating *Delta* transcription initiation is excluded. We then determined whether the pre-mRNA splicing efficiency of *Delta* was affected by reduction of dBCAS2. The three *Delta* mRNAs in *Drosophila* differ only in the 3’UTR and encode an identical protein [30] (GenBank: NT_033777.2). Therefore, three pairs of primers to detect the processed mRNA and two pairs of primers targeting the intron-containing precursor mRNA of *Delta* were designed (Fig 2D). To obtain RNA from *dBCAS2*-depleted tissues, the *Act5C-GAL4* driving *dBCAS2* ^*dsRNA*^ was generated. We then harvested RNA from the imaginal wing discs of *Act5C-GAL4* driving *dBCAS2* ^*dsRNA*^. The results were that, when *dBCAS2* ^*dsRNA*^ was expressed, the mRNA level of *Delta* decreased and pre-mRNA of *Delta* accumulated (Fig 2E, black bars). The increase in the pre-mRNA/mRNA ratio indicated that the pre-mRNA splicing efficiency of *Delta* decreased when *dBCAS2* was depleted. In addition, dBCAS2 could also rescue the reduced pre-mRNA splicing efficiency caused by lower expression of dBCAS2 (Fig 2E, grey bars); similar results also showed by *hBCAS2* rescue (S4 Fig). In conclusion, deprivation of *dBCAS2* indeed results in the down-regulation of Delta-Notch signaling through regulation of *Delta* pre-mRNA splicing.

### The *dBCAS2* ^*dsRNA*^ diminishing Delta-Notch activity is apoptosis-independent

**Fig 3:**
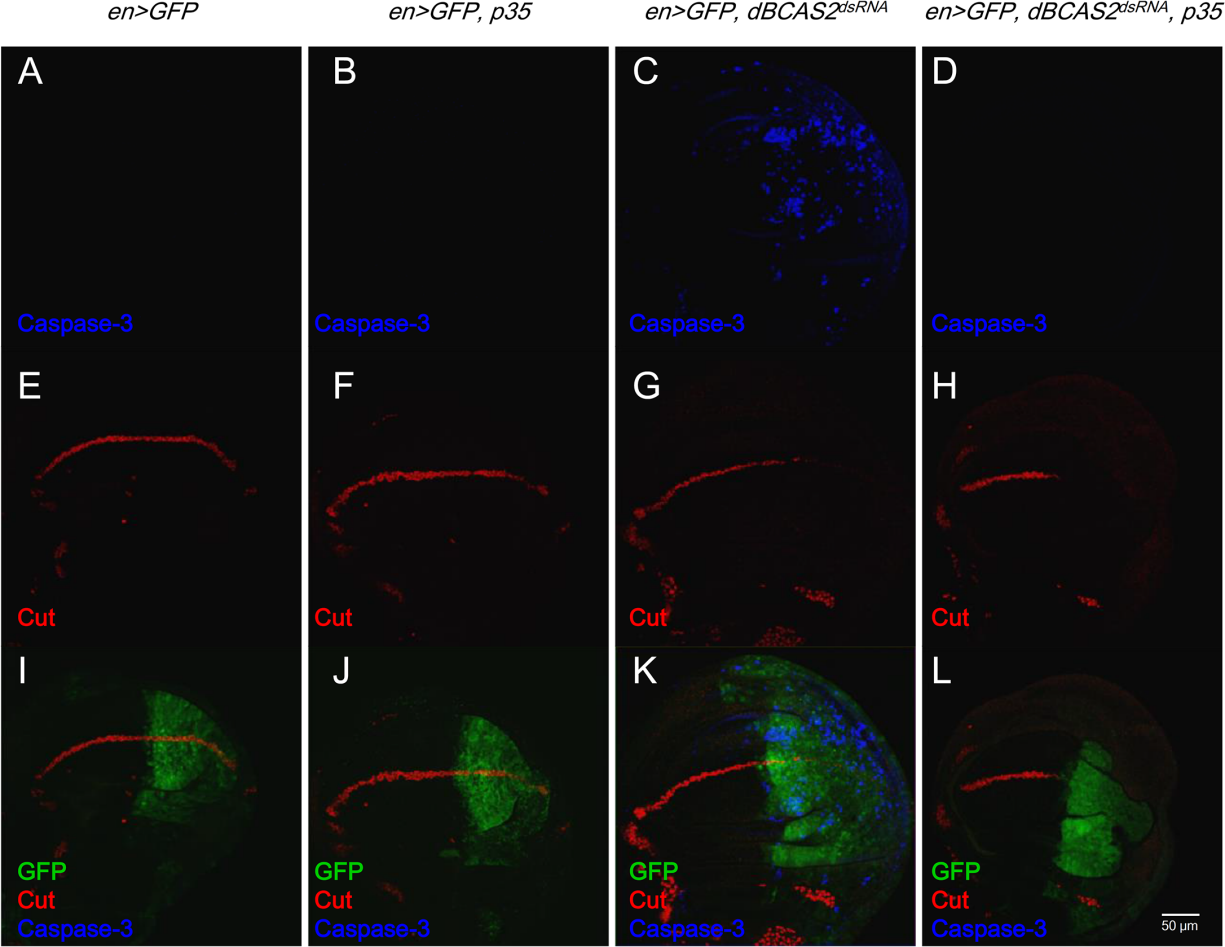
Deprivation of BCAS2 diminishing Delta-Notch activity is apoptosis-independent. The late third instar larval wing discs of each genotype were isolated and immunostained with anti-Caspase-3 antibody (blue; A-D); anti-Cut antibody (red, E-H); the expression of GFP, Cut and Caspase-3 were merged (I-L). Images were taken by confocal microscopy, scale bar, 50 μm. (A, E, I) Control (*en*>*GFP*). (B, F, J**)** The p35 over expression wing disc (*en>GFP*, *p35*). (C, G, K) The *dBCAS2*-depleted wing disc (*en*>*GFP*, *dBCAS2* ^*dsRNA*^). (D, H, L) The coexpression of p35 and *dBCAS2* ^*dsRNA*^ (*en*>*GFP*, *dBCAS2* ^*dsRNA*^, *p35*).

The deletion of BCAS2 in cells and fly can induce p53 expression [11] To rule out the apoptosis effect on the deprivation of BCAS2 could diminish Delta-Notch activity (Fig 1D, 1G, 1J and 1M), the apoptotic suppressor-*p35* expressed fly (*en*>*GFP*, *p35*) was crossed with *dBCAS2* ^*dsRNA*^ fly. The protein p35, a baculoviral protein, can inhibit caspase-3 activity to reduce cell apoptosis [31, 32]. Hence the caspase-3 acts as an indicator of apoptosis and Cut as a BCAS2-targeted gene expression. As shown in Fig 3, the expression patterns of caspase 3 and Cut in p35 fly (*en*>*GFP*, *p35*) (Fig 3B and 3F) showed the same as control (*en*>*GFP*) (Fig 3A and 3E). But when dBCAS2 expression was reduced in *dBCAS2* ^*dsRNA*^ fly, the expression of caspase-3 was observed; indicating that deprivation of BCAS2 indeed causes apoptosis (Fig 3C and 3G). However the fly (*en*>*GFP*, *dBCAS2* ^*dsRNA*^,*p35*) revealed the low caspase-3 that coupled with the decreased Cut level (Fig 3D and 3H) compared to control; implying that the decreased Cut expression in *dBCAS2*-depletion fly is not caused by cell death. In sum, the down regulation of Delta-Notch activity by deprivation of BCAS2 is apoptosis-independent, when both *p35* and *dBCAS2* ^*dsRNA*^ expressed.

### Overexpression of Delta can rescue the deformation of wing margin in *dBCAS2*-deprivation fly

**Fig 4:**
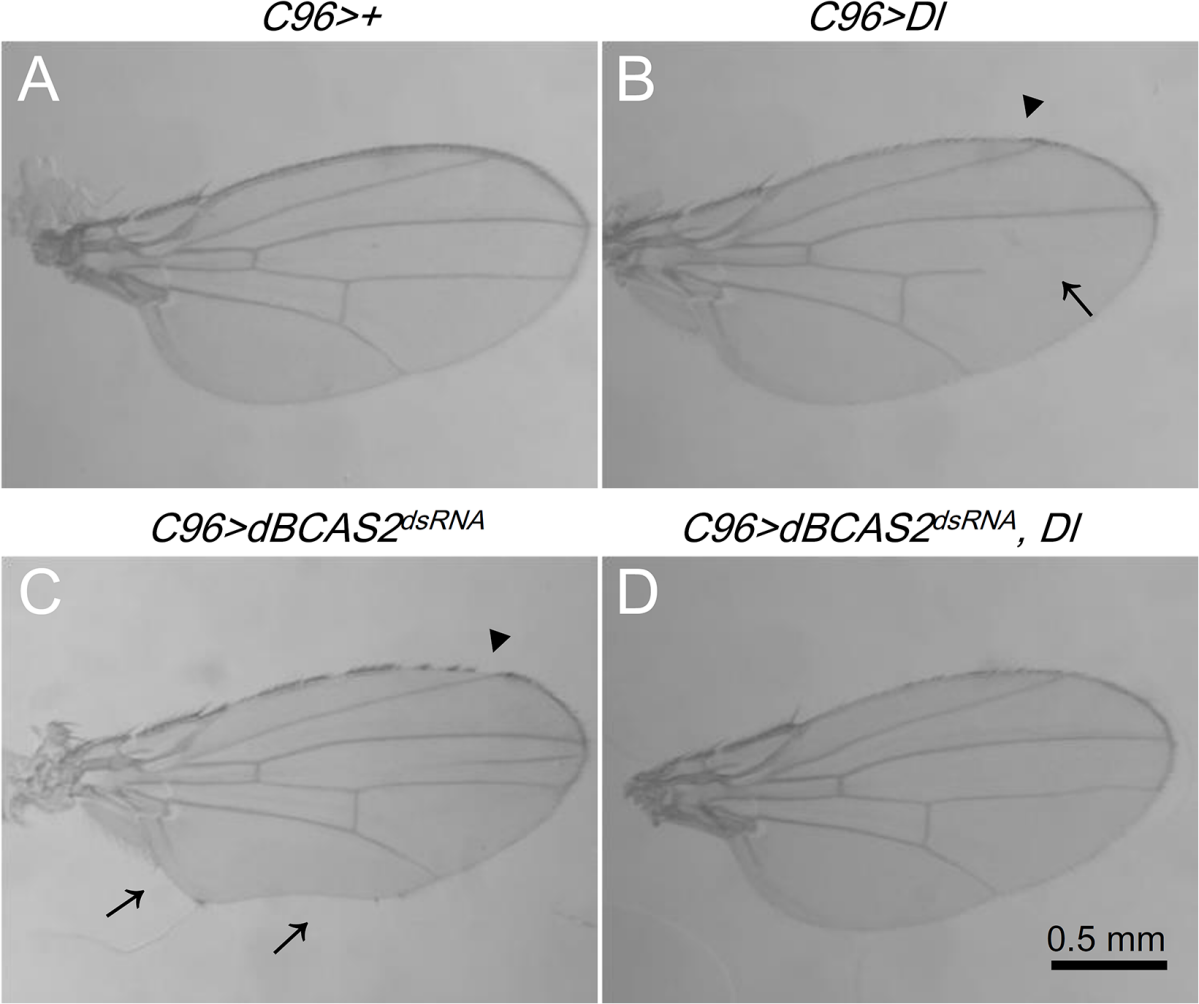
Overexpression of Delta can rescue the defected wing margin in deprived *dBCAS2* fly. The coexpression of *dBCAS2* ^*dsRNA*^ and *Dl* fly was generated under the control of *C96-GAL4*. (A) Control adult wing (*C96>+*). (B) Overexpression of *Delta* (*C96>Dl*). Arrow: sparse bristles; arrowhead: shortened wing vein. (C) The deformation of wing margin in (*C96>dBCAS2* ^*dsRNA*^). Arrowhead: sparse bristles; arrow: notched margin. (D) Overexpression of *Delta* with *dBCAS2* ^*dsRNA*^ (*C96 >dBCAS2* ^*dsRNA*^, *Dl*). Scale bar, 0.5 mm.

To investigate whether BCAS2 regulated *Delta* gene expression and in turn involved in fly wing development process, we generated the co-expressing *dBCAS2* ^*dsRNA*^ and *Dl* fly by crossing *C96 >Dl* with *dBCAS2* ^*dsRNA*^ fly. Due to the Delta gene in UAS-Dl is cDNA construct, there is no effect by dBCAS2^dsRNA^ splicing. Except for missing of stout mechanosensory organs and shortening of wing vein [33], the *Delta* overexpressing fly (*C96 >Dl)* showed the normal wing margin morphology (Fig 4B). As mentioned in Fig 1B, the adult *C96 >dBCAS2* ^*dsRNA*^ showed a notched wing margin (Fig 4C). But the wing margin morphology in fly co-expressing *dBCAS2* ^*dsRNA*^ and *Dl* was recovered to normal (Fig 4D), indicating that the *Delta* expression driven by *C96-GAL4* can compensate the reduced expression of endogenous *Delta* caused by deprivation of *dBCAS2* and thus rescue the wing margin morphology. Taken together, BCAS2 may regulate *Drosophila* wing formation through Delta-Notch signaling pathway.

### Depletion of *dBCAS2* can rescue the eye aberration resulted from overexpression of Delta

**Fig 5:**
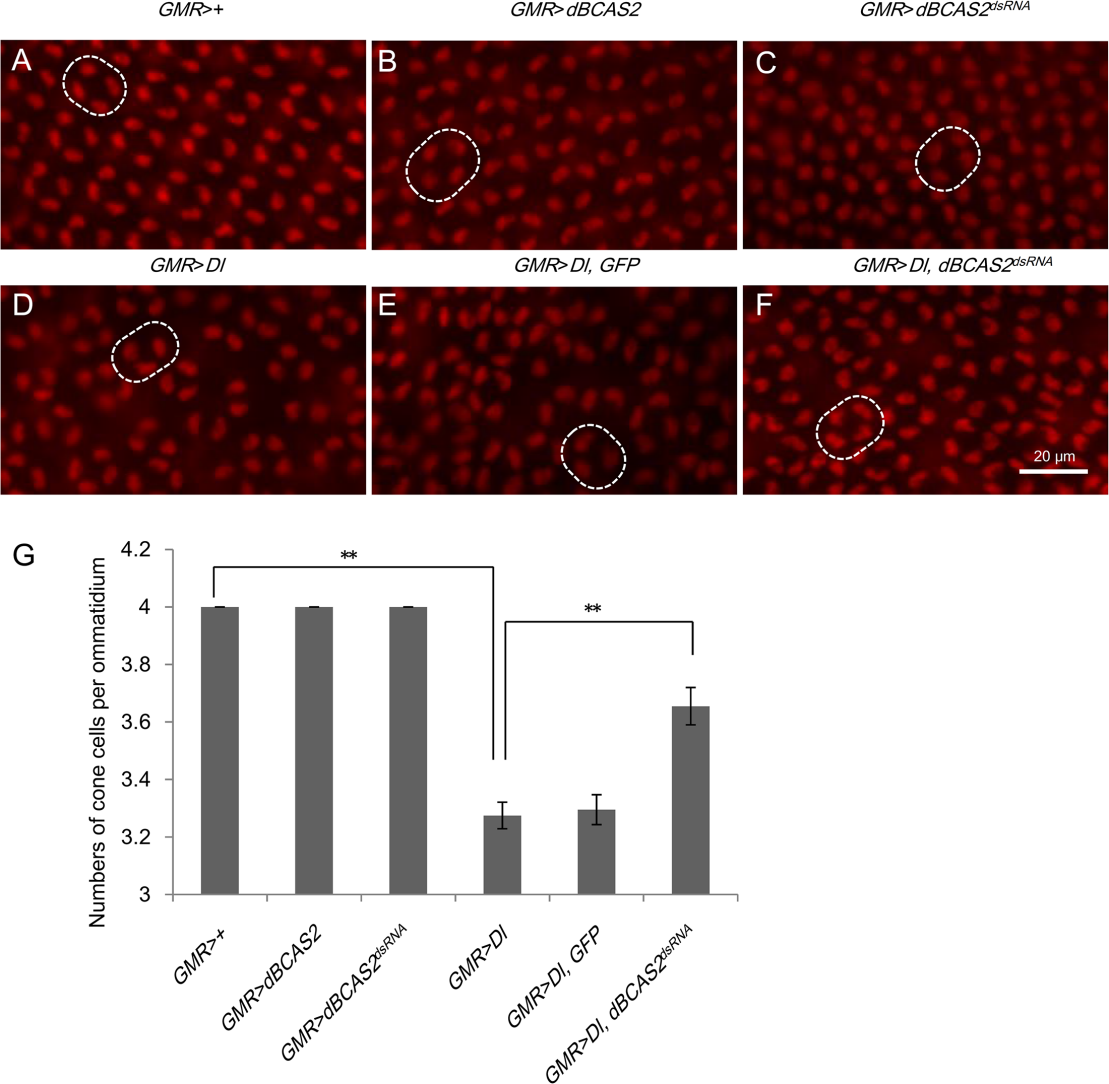
BCAS2 regulates the expression of Delta in developing eye. ***GMR*>*Dl* retinas reveal an aberrant phenotype.** After coexpression of *dBCAS2* ^*dsRNA*^ and *Dl*, the phenotype of *Dl*-overexpressing retinas could be rescued. Twenty-four hours after puparium formation (APF) retina were isolated and stained with anti-Cut antibody to analyze the formation of cone cells. Scale bar, 20 μm. (A) Retina in the control (*GMR*>*+*) eye. Control retina has an organized array of ommatidia and the average number of cone cells in each ommatidium is four. (B) *GMR*> *dBCAS2*. Ectopic expression of *dBCAS2* in retina (*GMR*>*dBCAS2*) resulted in the phenotypes similar to the control (A). (C) *GMR*>*dBCAS2* ^*dsRNA*^. The *dBCAS2*-depleted retina had a regular average number of cone cells per ommatidium, but exhibited disorganized and irregularly spaced ommatidia. (D) *GMR*>*Dl*. (E) *GMR*>*Dl*, *GFP* eye. (F) *GMR*>*Dl*, *dBCAS2* ^*dsRNA*^. The retinas of coexpression of *dBCAS2* ^*dsRNA*^ and *Dl* revealed more orderly array of ommatidia, compared to *GMR*>*Dl* retina. (G) Quantitation of the average number of cone cells per ommatidium. For cone cell analysis in each genotype, the number of cone cells per ommatidium (drawn by white dot line) was counted from 20 ommatidia per retina and total 10 retinas were measured; and statistically analyzed by unpaired two-tailed Student's t test **p<0.01.

To further characterize BCAS2-regulating *Delta*, we used *Delta*-overexpressing eye, a genetic background sensitive to the level of Delta, to elucidate this issue. Delta-Notch signaling is required for induction of cone cell and primary pigment cell fates in *Drosophila* eye development, a classic model for studying the mechanism of Delta-Notch signaling [34–36]. The organization of the ommatidial array and the average number of cone cells per ommatidium are taken as indicators of Delta-Notch signaling activity by staining with Cut antibody in after puparium formation (APF) retinas [36, 37]. When a high level of Delta is expressed only in photoreceptor cells by *Elav*-*GAL4*, the number of cone cells increases [38]. In contrast, when a high level of Delta is expressed in all types of differentiated cells in the eye by *GMR-GAL4*, the number of cone cells decreased, suggesting a dominant negative effect of the *GMR-GAL4* driven Delta in specifying the cone cell fate [33]. To confirm BCAS2-regulation of Delta-Notch signaling, we used this genetic approach to determine whether the depletion of *dBCAS2* in the *GMR*-driven, *Delta*-overexpressing eye could reduce total amount of Delta by reducing the endogenous Delta expression and thus rescue the number of cone cells [38, 39]. As shown in Fig 5, *GMR*>*dBCAS2* retinas (Fig 5B) revealed orderly ommatidia and four cone cells per ommatidium (normal average number) as for the control (Fig 5A). On the other hand, reduced dBCAS2 expression in the retinas (*GMR*>*dBCAS2* ^*dsRNA*^) (Fig 5C) led to a normal average number of cone cells per ommatidium but a disorganized array of ommatidia and irregular ommatidia spacing were observed. The retinas overexpressing *Delta* (*GMR*>*Dl)* had a lower number of cone cells per ommatidium, disorganized ommatidia and irregular ommatidia spacing, the average number of cone cells per ommatidium being 3.28 (Fig 5D and 5G). This is significantly lower than the constant number of four cone cells per ommatidium in control retina (Fig 5A) and indicates the down-regulation of Delta-Notch signaling activity, similar to a previous report[33]. However, the organized ommatidia and four cone cells per ommatidium were restored in the *GMR*>*Dl*, *dBCAS2* ^*dsRNA*^ retina (Fig 5F) from the disordered phenotypes of the *GMR*>*Dl* retina (Fig 5D). To confirm that the rescue effect of dBCAS2^dsRNA^ in the *Dl*-overexpressing retina was not the result of a dilution effect of the *GAL4* activator, the *UAS-GFP*; *GMR-GAL4* strain was crossed with *UAS-Dl* to simultaneously express *Delta* and *GFP* (*GMR*>*Dl*, *GFP*). As shown in Fig 5E, the *GMR*>*Dl*, *GFP* retina had a similar phenotype to that of the *GMR*>*Dl* retina (Fig 5D). These results provide additional evidence that dBCAS2 is involved in the regulation of Delta-Notch signaling in the fly, through the regulation of *Delta* pre-mRNA splicing.

## Discussion

In this study, we show that BCAS2 is involved in regulating *Delta* pre-mRNA splicing, which then affects the Delta-Notch signaling and causes wing deformation. The decreased BCAS2 expression in *Drosophila* reduces Delta and Notch expression and Notch target genes, [*E(spl)*]bHLH and Cut. The reduced expression of Delta-Notch signaling related genes is correlated with wing deformation and the impairment of *Delta* pre-mRNA splicing function but not with the initiation of transcription of *Delta* (Figs 1 and 2). In addition, BCAS2 overexpression can not only rescue the wing damage phenotype induced by depletion of BCAS2 but also restore *Delta* pre-mRNA splicing and Delta-Notch signaling activity (Fig 1 and S3 Fig), consistent with previous reports which demonstrated that the aberrant Delta-Notch signaling can lead to abnormal wing development [22].

BCAS2-deprived cell can cause apoptosis due to the p53 activation [11, 40]. To exclude the apoptosis-causing declination of Delta-Notch signaling, the apoptosis inhibitor-*p35* was expressed simultaneously with *BCAS2* deprivation in fly (*en*>*GFP*, *dBCAS2* ^*dsRNA*^, *p35*). Even though the apoptosis effect was eliminated, the Delta-Notch signaling still declined. It indicates that the decreased Delta-Notch signaling caused by BCAS2-deprivation is apoptosis-independent (Fig 3). In addition, the wing margin morphology defection in depletion of *dBCAS2* can be recovered to normal by *Delta*-overexpressing (Fig 4); plus depletion of *dBCAS2* can restore the aberrant eye associated with *Delta*-overexpressing retinas (Fig 5); providing supporting evidences for the regulation of Delta-Notch signaling by BCAS2.

BCAS2 can regulate Delta-Notch signaling, which is a developmental signaling pathway conserved through evolution and is involved in cell fate determination and patterning interaction in *Drosophila* [19]. Abnormal Delta-Notch signaling can lead to early embryonic lethality [41, 42] and may be the reason for the lethality of the *Drosophila* BCAS2 ubiquitously deprived fly [11]. In this study, we used the *Drosophila* adult wing and imaginal wing disc to investigate the effect of BCAS2-induction on Delta-Notch signaling, which is involved in the development of the adult wing, hinge and notum through lateral inhibition, lineage decisions and boundary formation [43]. Within a wing disc, compartments and wing primordium are formed under the expression regulation of selector genes, including posterior-specific transcription factor *engrailed* (*en*), dorsal-specific transcription factor *apterous* (*ap*) and *vestigial* (*vg*), which defines the region of wing primordium (wing pouch). The temporally and spatially precise expressions of selector genes and the developmental signaling molecules, including Notch (N), Decapentaplegic (Dpp), Wingless (Wg) and Hedgehog (Hh), make up the delicate adult wing. The formation of wing margin and veins also requires the activity of Delta-Notch signaling on dorsal/ventral (D/V) boundary and provein cells in the fly wing primordium, respectively [20, 43]. To study wing morphology in this and our previous study, we used *ms1096-GAL4*, which is expressed in the pouch region of wing discs [29], to drive the expression of *dBCAS2* ^*dsRNA*^ to reduce dBCAS2 expression. All of the progeny carrying *UAS-dBCAS2* ^*dsRNA*^ driven by *ms1096-GAL4* exhibits twisted and shrunken wings (S1Cb Fig). However, for clear illustration and comparison of molecular mechanisms regulated by BCAS2, we used *engrailed-GAL4* to drive the expression of *dBCAS2* ^*dsRNA*^ in the posterior compartment of the wing disc in third instar larvae. The results illustrate an obvious defect of Delta-Notch signaling activity in the posterior part, but not in the anterior part, this being correlated with the depletion of *BCAS2* expression (Fig 1).

On the other hand, ommatidial development in the *Drosophila* compound eye is paradigm for addressing the molecular mechanism of lateral inhibition, the prominent characteristic of Delta-Notch signaling. Delta-Notch interaction within the same cell leads to *cis*-inhibition of Notch by Delta [44]. Because the differentiation of cone cells requires the activation of Delta-Notch signaling [45], a reduction of Delta-Notch signaling causes a decrease in the number of cone cells per ommatidium [33, 44, 46, 47]. Hence, the Delta-overexpressing fly exhibits an aberrant retina with a lower number of cone cells, but this aberration can be rescued to normal in cone cell number and organized ommatidia when the *Delta*-overexpressing fly is crossed with the *dBCAS2* ^*dsRNA*^ transgenic fly (Fig 5). The results of these two systems, wing (Fig 4) and eye (Fig 5), corroborate further that BCAS2 regulates Delta-Notch signaling. In addition to Delta-Notch signaling, Dpp, Wg, and Hh also are essential for the temporally and spatially precise expression of the developmental signaling molecules to make up the delicate adult wing and eye [48].

Here we show that BCAS2 regulates Delta-Notch signaling. It will be worth for further investigating the specificity or dose effect of Delta-Notch signaling-induced phenotypes by BCAS2. Delta-Notch signaling is initially derived from ligand Delta of one cell interacting with Notch of the neighbor cell and in turn Notch can be cleavage into NCID. Then the cytoplasm NCID translocates into nucleus to stimulate the targeted genes’ expression in a variety of context. Hence the regulation of Delta-Notch signaling can be happened on several different levels those remain unanswered questions; for examples, the ligand activation effect, Notch receptor cleavage proteins, Notch receptor down-regulation etc [49–52]. Hence, the different reducing amount of endogenous BCAS2 via RNAi and the threshold declination level of BCAS2 in fly can affect Delta-Notch signaling that resulted in the wing deformation those will be interesting for further examination. Moreover, BCAS2 is a member of hPrp19 complex and functions for pre-mRNA splicing. It still needs to answer whether BCAS2 is specific for *Delta* gene splicing or there are more candidate genes to be targeted. Further comprehensive investigation will be required to understand whether other developmental related genes are regulated by BCAS2.

## Materials and Methods

### Fly genetics and fly stocks

*Drosophila* stocks were kept and crossed at 25°C and supplied with cornmeal medium. The generation of *UAS-dBCAS2* ^*dsRNA*^ */T (2; 3) SM6-TM6B*, *ms1096-GAL4*, *Act5C-GAL4*, *engrailed (en)-GAL4*, and *w* ^*1118*^ had been described previously [11]. *C96-GAL4* (stock no. 257575), *GMR-GAL4* (stock no. 27617), *UAS-Dl-LacZ* ^*05151*^ (stock no. 11651), *UAS-m8-LacZ* (stock no. 26786), *UAS-p35* (stock no. 5073), and *UAS-Delta* (stock no. 26695) were obtained from Bloomington *Drosophila* Stock Center and maintained by the Fly Core Facility in the College of Medicine, National Taiwan University. The *UAS-dBCAS2* ^*dsRNA*^ was purchased from Vienna *Drosophila* RNAi Center (stock no. 26676) and balanced over *T(2;3)SM6-TM6B*, a translocated chromosome 2–3 balancer [11]. The *UAS-dBCAS2* was generated in Taiwan Fly Core by microinjection of pUAST-dBCAS2 using the standard procedure as *UAS-hBCAS2* strain described previously [11]. The *UAS-dBCAS2* ^*dsRNA*^, *UAS-dBCAS2*/*T (2;3) SM6-TM6B* was generated by recombination of *UAS-dBCAS2* ^*dsRNA*^ with *UAS-dBCAS2* balancing over *T (2;3) SM6-TM6B (14*); either the *UAS-hBCAS2* ^*dsRNA*^, *UAS-dBCAS2*/*T (2;3) SM6-TM6B*, was generated. The *UAS-dBCAS2* ^*dsRNA*^; *Dl-LacZ* ^*05151*^/*T (2;3) SM6-TM6B* was established by balancing *UAS-dBCAS2* ^*dsRNA*^ and *Dl-LacZ* ^*05151*^, on chromosome II and III respectively, over *T (2;3) SM6-TM6B*, enabling co-segregation of *UAS-dBCAS2* ^*dsRNA*^ and *Dl-LacZ* ^*05151*^ in offspring. *The UAS-dBCAS2* ^*dsRNA*^; *UAS-Dl*/*T (2;3) SM6-TM6B* was generated with similar method as that was used to generate *UAS-dBCAS2* ^*dsRNA*^; *Dl-LacZ* ^*05151*^/*T (2;3) SM6-TM6B*.

### Plasmid construction

The dBCAS2 coding sequence (CDS) from a *Drosophila* S2 cell cDNA library was amplified using the polymerase chain reaction (PCR) and cloned into the pOSI-T vector (pOSI-T PCR Cloning Kit, GeneMark). *UAS-3xFLAG-dBCAS2* strain was generated by insertion of the dBCAS2 CDS into pUAST-3xFLAG vector. The XhoI and XbaI sites were added to the 5’ and 3’ ends, respectively, of the dBCAS2 CDS before insertion into the pUAST-3xFLAG vector. The constructed pUAST-3xFLAG-dBCAS2 was checked by sequencing. Primers used to construct pUAST-3xFLAG-dBCAS2 are listed in S2 Table.

### Immunofluorescence

The wing imaginal discs and pupal retinas were dissected from late third instar larvae and twenty-four hours after puparium formation (APF) pupae, respectively, then fixed for 17 min in phosphate-buffered saline (PBS) with 4% paraformaldehyde and then blocked in PBS with 0.3% Triton X and 5% BSA (bovine serum albumin) for 30 min at room temperature and incubated with primary antibodies overnight at 4°C. After washed with PBS-Triton X three times, the wing discs were incubated with secondary antibody for 1 h at room temperature. The stained wing discs were then mounted on the slides with glycerol. Primary antibodies: mouse anti-Cut (2B10, 1:200, Developmental Studies Hybridoma Bank (DSHB), Iowa City, IA, USA), mouse anti-Delta (C594.B9, 1:200, DSHB), mouse anti-Notch (C17.9C6, 1:100, DSHB), mouse anti-β-gal (1:1000, Sigma-Aldrich, St. Louis, MO, USA), rabbit anti-cleaved caspase-3 (1:200; Abcam). Secondary antibodies [anti-mouse Cy3 (1:1000), anti- rabbit Cy5 (1:1000)] were purchased from Jackson ImmunoResearch Laboratories (West Grove, PA, USA). The fluorescent images were acquired by confocal microscope TCS SP5 (Imaging Core, First Core Labs, National Taiwan University College of Medicine) and *Axio Imager A1* Microscope (Zeiss, Thornwood, NY, USA). Leica LAS AF Lite and Adobe Photoshop were used to analyze and process images.

### Adult wing image processing and analysis

The phenotype of adult wings were examined by immersed adult flies in 100% isopropanol for at least 24 hours, then the wings were isolated and mounted on slides with Hoyer’s mounting medium (50 cc distilled water, 30 g gum arabic (U. S. P. Flake), 200 g chloral hydrate, 20 cc glycerin) [53]. Images of adult wing were obtained by using Dino-Lite Digital Microscope.

### In vivo splicing assays

For the detection of mRNA and pre-mRNA in *Drosophila*, total RNA from imaginal wing discs in 20 third instar larvae was isolated by using TRI reagent (Sigma-Aldrich). 1 microgram of total RNA was treated with DNase (RQ1, Promega, Madison, WI, USA) before reverse transcription (SuperScript III, Invitrogen). The cDNA was synthesized using random hexamers (Invitrogen) and oligo dT (Invitrogen) for detection of pre-mRNA and mRNA respectively. Quantitative RT-PCR (qRT-PCR) analyses (KAPA SYBR Fast, KAPA Biosystem, Woburn, MA, USA,) were performed according to the manufacturer’s instructions. Applied Biosystems 7500 Real-Time PCR System quantified the amount of mRNA and pre-mRNA. To determine the amount of *Delta* pre-mRNA, primers of *Delta* I3-E4 were targeted to the sequence spanning from the 3’ end of intron 3 to the 5’ end of exon 4; *Delta* I5 recognized the sequence within intron 5 (Fig 2C). To measure the amount of *Delta* mRNA, primers of *Delta* E2-E3 amplified the sequence spanning from the 3’ end of exon 2 to the 5’ end of exon 3; *Delta* E2-E4 targeted to the sequence extending across from the 3’ end of exon 2 to the 5’ end of exon 4; *Delta* E4-E5 amplified the sequence spanning from the 3’ end of exon 4 to the 5’ end of exon 5. Other primer sequences are given in S2 Table.

### Western Blotting

The cell lysates from the late third instar larvae were harvested and performed western blotting with mouse anti-FLAG M2 (1:10000, Sigma-Aldrich), rabbit anti-hBCAS2 (1: 10000, Bethyl Laboratories), mouse anti-tubulin (1:10000, Calbiochem, San Diego, CA, USA), and mouse anti-Actin (Sigma); separately.

## Supporting Information

S1 Fig The *dBCAS2* transgenic fly shows normal wing morphology. The *ms1096-GAL4* was used to drive the ectopic expression of *dBCAS2*, whose 5’ end was tagged with 3xFLAG. (a) Control adult wing (*ms1096*>*+)*. (b) The *dBCAS2* transgenic fly (*ms1096*>*dBCAS2*). (B) BCAS2 protein expression in *dBCAS2* transgenic flies. BCAS2 protein was analyzed from the body extract of third instar larvae by western blot with anti-Flag antibody. (C) Coexpression of *dBCAS2* ^*dsRNA*^ and *dBCAS2*, driven by *ms1096-GAL4*, yields a rescued wing that resembles the control. (Ca) Control wing; (Cb) The *dBCAS2*-depleted wing (*ms1096*>*dBCAS2* ^*dsRNA*^); (Cc) The rescued wing (*ms1096*>*dBCAS2* ^*dsRNA*^, *dBCAS2*). Scale bar, 0.5 mm.
(TIF)

S2 Fig The *hBCAS2* transgenic fly shows normal Delta-Notch signaling. (A, C, E) Control (*en*>*GFP)*. (B, D, F) The *hBCAS2* transgenic fly (*en*>*GFP*, *hBCAS2*). Wing discs were stained with the indicated antibody. The images were taken by fluorescent microscope. The expression of Delta (red), Notch (white in the left panel; red in the right panel), and Cut (red) in the GFP-marked posterior compartment of wing discs could be observed. (A, B) Delta; (C, D) NICD; (E, F) Cut. Scale bar 50 μm.
(TIF)

S3 Fig The hBCAS2 can rescue the elimination of Delta, Notch and Cut expression caused by the depletion of dBCAS2. (A, D, G) Control (en>GFP). (B, E, H) The dBCAS2 depleted wing discs (en>GFP, dBCAS2^dsRNA^). (C, F, I) The Rescued wing discs (en>GFP, dBCAS2^dsRNA^, hBCAS2). (A, B, C) Anti-Delta antibody; (D, E, F) Anti-NICD antibody; (G, H, I) Anti-Cut antibody. The expression of (C) Delta (red), (F) Notch (red) and (I) Cut (red) in the GFP-marked posterior compartment of wing discs could be rescued.
(TIF)

S4 Fig RT-PCR analysis of *Delta* pre-mRNA and mRNA in coexpression of *hBCAS2* and *dBCAS2* ^*dsRNA*^. Coexpression of *hBCAS2* and *dBCAS2* ^*dsRNA*^ (gray bar) could rescue the phenotypes of *Delta* pre-mRNA splicing inefficiency in *dBCAS2*-depleted larvae (black bar). Data are shown as means and SD relative to the controls from three independent experiments. The p-values was measured by the Student’s t-test. **p<0.01.
(TIF)

S1 Table Genotypes of *Drosophila* analyzed. (DOCX)

S2 Table Primer sequences used in this study. (DOCX)
