# Supplementary figures and images for: BCAS2 Regulates Delta-Notch Signaling Activity through Delta Pre-mRNA Splicing in Drosophila Wing Development
Source: PLoS One. 2015 Jun 19;10(6):e0130706. doi: 10.1371/journal.pone.0130706 (PMC4475048; doi:10.1371/journal.pone.0130706)

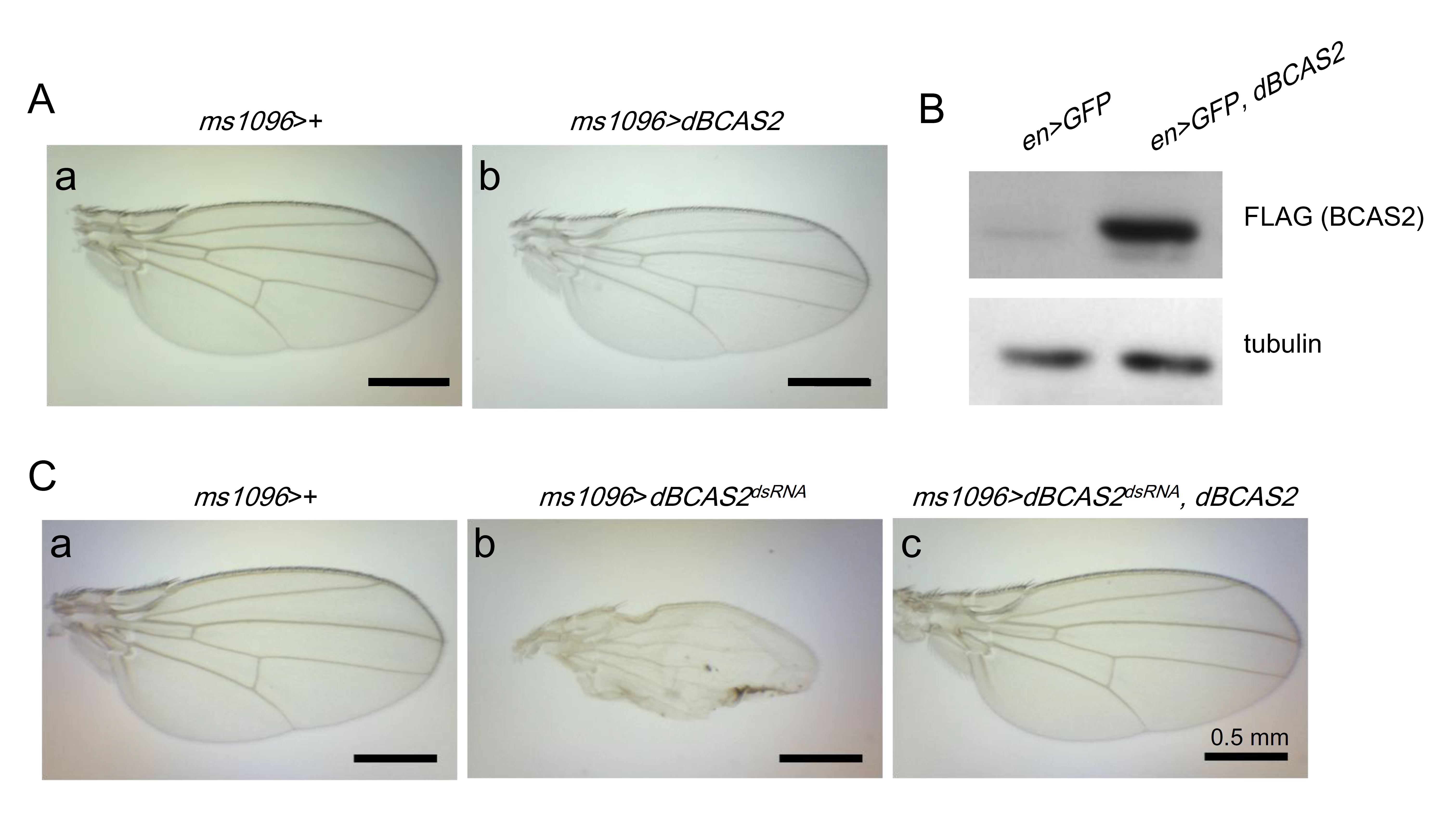

Supplement: S1 Fig — The ms1096-GAL4 was used to drive the ectopic expression of dBCAS2, whose 5’ end was tagged with 3xFLAG. (a) Control adult wing (ms1096>+). (b) The dBCAS2 transgenic fly (ms1096>dBCAS2). (B) BCAS2 protein expression in dBCAS2 transgenic flies. BCAS2 protein was analyzed from the body extract of third instar larvae by western blot with anti-Flag antibody. (C) Coexpression of dBCAS2 dsRNA and dBCAS2, driven by ms1096-GAL4, yields a rescued wing that resembles the control. (Ca) Control wing; (Cb) The dBCAS2-depleted wing (ms1096>dBCAS2 dsRNA); (Cc) The rescued wing (ms1096>dBCAS2 dsRNA, dBCAS2). Scale bar, 0.5 mm. (TIF) [file pone.0130706.s001.tif]

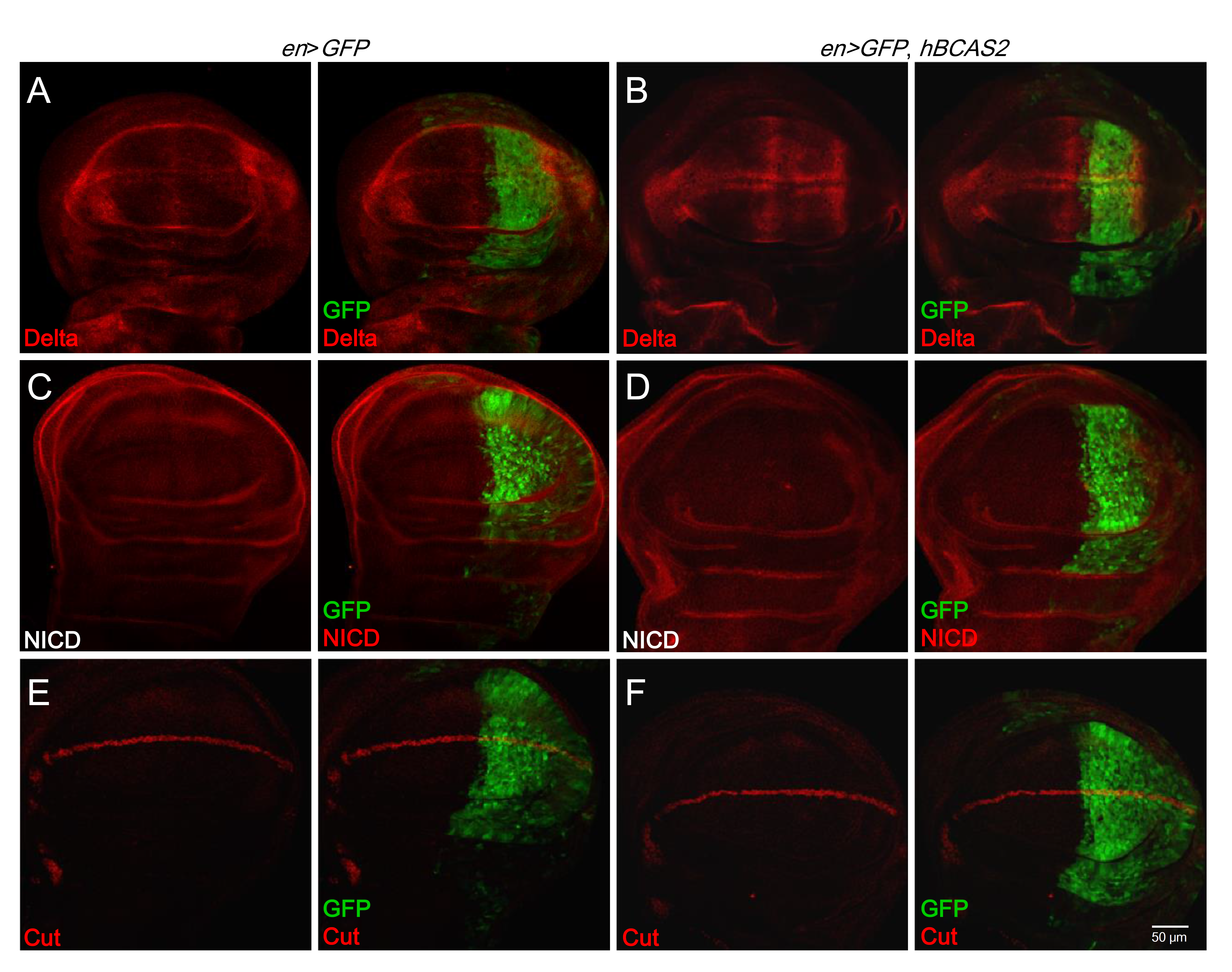

Supplement: S2 Fig — (A, C, E) Control (en>GFP). (B, D, F) The hBCAS2 transgenic fly (en>GFP, hBCAS2). Wing discs were stained with the indicated antibody. The images were taken by fluorescent microscope. The expression of Delta (red), Notch (white in the left panel; red in the right panel), and Cut (red) in the GFP-marked posterior compartment of wing discs could be observed. (A, B) Delta; (C, D) NICD; (E, F) Cut. Scale bar 50 μm. (TIF) [file pone.0130706.s002.tif]

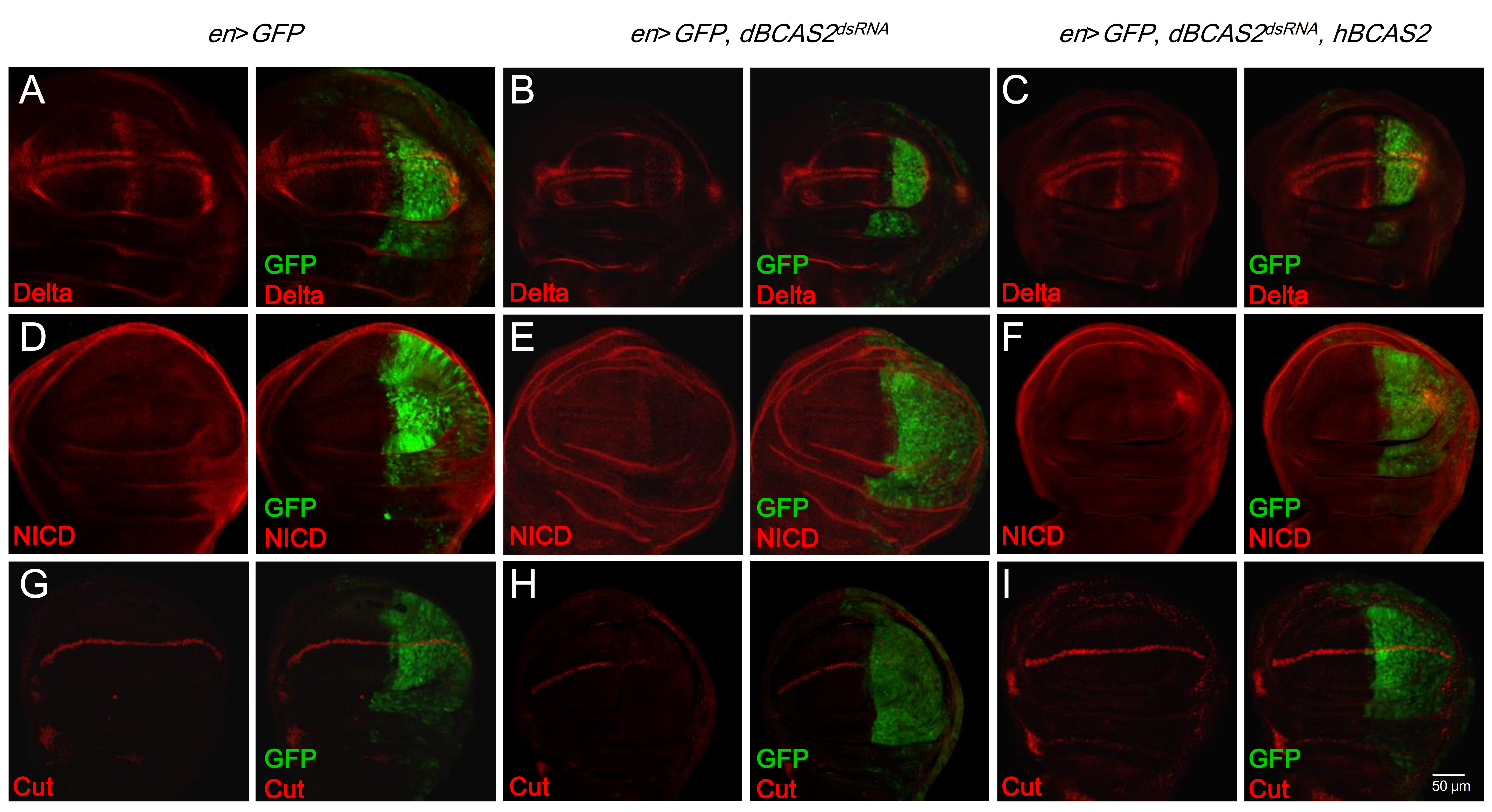

Supplement: S3 Fig — (A, D, G) Control (en>GFP). (B, E, H) The dBCAS2 depleted wing discs (en>GFP, dBCAS2dsRNA). (C, F, I) The Rescued wing discs (en>GFP, dBCAS2dsRNA, hBCAS2). (A, B, C) Anti-Delta antibody; (D, E, F) Anti-NICD antibody; (G, H, I) Anti-Cut antibody. The expression of (C) Delta (red), (F) Notch (red) and (I) Cut (red) in the GFP-marked posterior compartment of wing discs could be rescued. (TIF) [file pone.0130706.s003.tif]

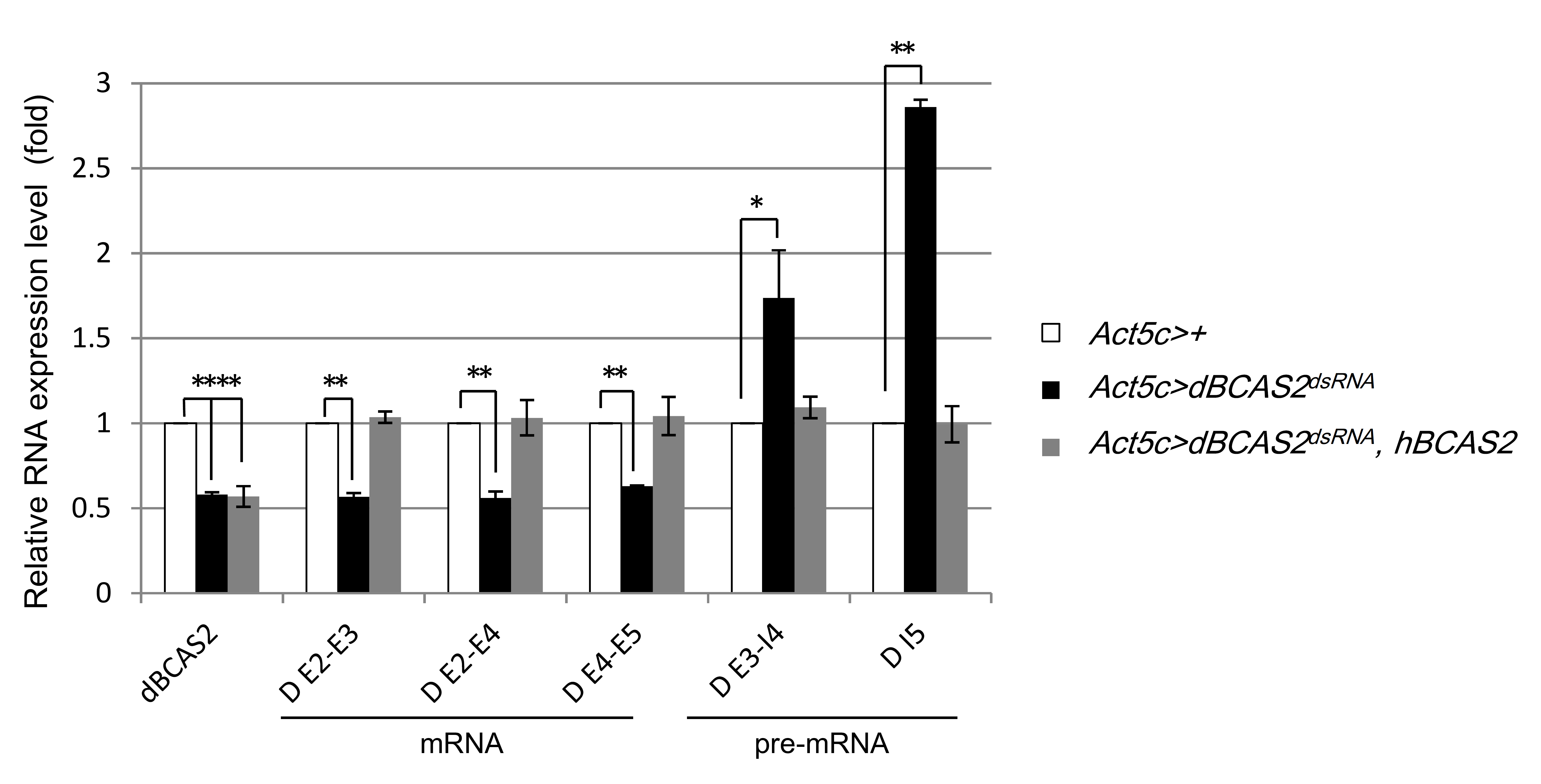

Supplement: S4 Fig — Coexpression of hBCAS2 and dBCAS2 dsRNA (gray bar) could rescue the phenotypes of Delta pre-mRNA splicing inefficiency in dBCAS2-depleted larvae (black bar). Data are shown as means and SD relative to the controls from three independent experiments. The p-values was measured by the Student’s t-test. **p<0.01. (TIF) [file pone.0130706.s004.tif]
