# Supplementary material for: BCAS2 Regulates Delta-Notch Signaling Activity through Delta Pre-mRNA Splicing in Drosophila Wing Development
Source: PLoS One. 2015 Jun 19;10(6):e0130706. doi: 10.1371/journal.pone.0130706 (PMC4475048; doi:10.1371/journal.pone.0130706)
Supplement: S1 Table — (DOCX) [file pone.0130706.s005.docx]

**Supplementary Table S1**. Analyzed genotypes of *Drosophila*

| For immunohistochemistry and observation of adult wings | | | | |
| --- | --- | --- | --- | --- |
| Analyzed tissue | | | Denoted as | Genotype |
| Adult wing/Wing discs | Control | | *C96*>+ | *C96-GAL4*/+ |
|  |  |  | *en*>*GFP* | *en-UAS-GFP, GAL4*/+ |
|  |  |  | *ms1096*>+ | *ms1096-GAL4*/+ |
|  | dBCAS2-depletion | | *C96*>*dBCAS2^dsRNA^* | *UAS-dBCAS2^dsRNA^*/+; *C96-GAL4*/+ |
|  |  |  | *en*>*GFP*, *dBCAS2^dsRNA^* | *en-UAS-GFP, GAL4*/*UAS-dBCAS2^dsRNA^* |
|  |  |  | *ms1096*>*dBCAS2^dsRNA^* | *ms1096-GAL4*/+; *UAS-dBCAS2^dsRNA^*/+ |
|  | 3xFLAG-dBCAS2 overexpression | | *ms1096*>*dBCAS2* | *ms1096-GAL4*/+; *UAS*-*dBCAS2*/+ |
|  |  |  | *en*>*GFP*, *dBCAS2* | *en-UAS-GFP, GAL4*/*UAS-dBCAS2* |
|  | hBCAS2 overexpression | | *en*>*GFP*, *hBCAS2* | *en-UAS-GFP, GAL4/UAS-hBCAS2* |
|  | dBCAS2-depletion rescued with ectopic expression of 3xFLAG~~-~~dBCAS2 | | *ms1096*>*dBCAS2^dsRNA^*, *dBCAS2* | *ms1096-GAL4*/+; *UAS-dBCAS2^dsRNA^*, *UAS-3xFLAG-dBCAS2*/+ |
|  |  |  | *en*>*GFP*, *dBCAS2^dsRNA^*, *dBCAS2* | *UAS-GFP, en-GAL4*/*UAS-dBCAS2^dsRNA^*, *UAS~~-~~dBCAS2* |
|  | dBCAS2-depletion rescued with ectopic expression of hBCAS2 | | *en*>*GFP*, *dBCAS2^dsRNA^,* *hBCAS2* | *en-UAS-GFP, GAL4*/*UAS-dBCAS2^dsRNA^, UAS-hBCAS2* |
|  | p35 overexpression | | *en>GFP, p35* | *en-UAS-GFP, GAL4/+*; *UAS-p35/+* |
|  | dBCAS2-depletion with p35 overexpression | | *en>GFP, dBCAS2^dsRNA^, p35* | *en-UAS-GFP, GAL4*/*UAS-dBCAS2^dsRNA^*; *UAS-p35/+* |
|  | Dl overexpression | | *C96>Dl* | *C96-GAL4/UAS-Dl* |
|  | dBCAS2-depletion with Dl overexpression | | *C96>dBCAS2^dsRNA^, Dl* | *UAS-dBCAS2^dsRNA^/+; C96-GAL4/UAS-Dl* |
|  | E(spl)m8-lacZ reporter | Control | *en*>*GFP* | *E(spl)m8-lacZ*/+; *UAS-GFP,* *en-GAL4*/+ |
|  |  | dBCAS2-depletion | *en*>*GFP*, *dBCAS2^dsRNA^* | *E(spl)m8-lacZ*/+; *UAS-GFP, en-GAL4*/*UAS-dBCAS2^dsRNA^* |
|  | Dl-lacZ reporter | Control | *en*>*GFP* | *en-UAS-GFP, GAL4*/+; *Dl-lacZ*/+ |
|  |  | dBCAS2-depletion | *en*>*GFP*, *dBCAS2^dsRNA^* | *UAS-GFP, en-GAL4* /*UAS-dBCAS2^dsRNA^*; *Dl-lacZ*/+ |
| Pupal retina | Control | | *GMR*>*+* | *GMR-GAL4*/+ |
|  | 3xFLAG-dBCAS2 overexpression | | *GMR*>*dBCAS2* | *GMR-GAL4*/*UAS-dBCAS2* |
|  | dBCAS2-depletion | | *GMR*>*dBCAS2^dsRNA^* | *GMR-GAL4*/*UAS-dBCAS2^dsRNA^* |
|  | Dl overexpression | | *GMR*>*Dl* | *GMR-GAL4*/+; *UAS*-*Dl*/+ |
|  | Dl overexpression rescued with depletion of dBCAS2 | | *GMR*>*Dl*, *dBCAS2^dsRNA^* | *GMR-GAL4*/*UAS*-*dBCAS2^dsRNA^*; *UAS*-*Dl*/+ |
|  | Coexpression of Dl and GFP | | *GMR*>*Dl*, *GFP* | *UAS-GFP*/+;*GMR-GAL4*/+; *UAS*-*Dl*/+ |

| For quantitative RT-PCR analysis | | | |
| --- | --- | --- | --- |
| CNSs and imaginal discs | Control | *Act5C*>+ | *Act5C-GAL4*/+ |
|  | dBCAS2-depletion | *Act5C*>*dBCAS2^dsRNA^* | *Act5C-GAL4*/*UAS*-*dBCAS2^dsRNA^* |
|  | dBCAS2-depletion rescued with ectopic expression of hBCAS2 | *Act5C*> *dBCAS2^dsRNA^,* *hBCAS2* | *Act5C-GAL4*/*UAS*- *dBCAS2^dsRNA^,* *UAS*-*hBCAS2* |
|  | dBCAS2-depletion rescued with ectopic expression of 3xFLAG-dBCAS2 | *Act5C*>*dBCAS2^dsRNA^*, *dBCAS2* | *Act5C-GAL4*/*UAS*- *dBCAS2^dsRNA^,* *UAS*-*dBCAS2* |
