# Supplementary material for: BCAS2 Regulates Delta-Notch Signaling Activity through Delta Pre-mRNA Splicing in Drosophila Wing Development
Source: PLoS One. 2015 Jun 19;10(6):e0130706. doi: 10.1371/journal.pone.0130706 (PMC4475048; doi:10.1371/journal.pone.0130706)
Supplement: S2 Table — (DOCX) [file pone.0130706.s006.docx]

**Supplementary Table S2.** Primer sequences used in this study.

| Plasmid Construction | |
| --- | --- |
| Clon-dBCAS2-F-1 | 5’-ATGGCTGGCGAAGTTATTGT-3’ |
| Clon-dBCAS2-R-837 | 5’-CTACGTTGATCCATTGGAAG-3’ |
| dBCAS2-XhoI | 5’-AAACTCGAGATGGCTGGCGAAGTTATTGT-3’ |
| dBCAS2-XbaI | 5’-AAATCTAGACTACGTTGATCCATTGGAAG-3’ |
| pUAST-N3 | 5’-TTCGTCTACGGAGCGACAAT-3’ |
| pUAST-C3 | 5’-TAAAGGCATTCCACCACTGC-3’ |
| Quantitative RT-PCR | |
| Histone 3 F | 5’-GTGAAGTAGTGAACGTGAAC-3’ |
| Histone 3 R | 5’-CCGCCGAGCTCTGGAATCGC-3’ |
| Rp49 F | 5’-CCAAGATCGTGAAGAAGCG-3’ |
| Rp49 R | 5’-GTTGGGCATCAGATACTGTC-3’ |
| dBCAS2 F | 5’-GCTCCAGCAATCCAGATCCA-3’ |
| dBCAS2 R | 5’-CTGTCACCATCGGCATCTGT-3’ |
| *Delta* E2-E3 F | 5’-CGCGTCTGCCTAAAGCACTAC-3’ |
| *Delta* E2-E3 R | 5’-GGTACCCGGCCATGAGAAC-3’ |
| *Delta* E2-E4 F | 5’-CATCCAGTTCCCCTTCTCGTT-3’ |
| *Delta* E2-E4 R | 5’-TCGGAGGACACTTCCAGTACCT-3’ |
| *Delta* E4-E5 F | 5’-CGGAGACGGGCGAAATTAT-3’ |
| *Delta* E4-E5 R | 5’-GGCTTGTCGCAATGTCCAT-3’ |
| *Delta* I3-E4 F | 5’-GTCTCGATCTAATCGCCGTCTT-3’ |
| *Delta* I3-E4 R | 5’-TCGTACTCCAGCGACGTGTACT-3’ |
| *Delta* I5 F | 5’-GATGCGTGGAACAGGTGATG-3’ |
| *Delta* I5 R | 5’-TGAAATGCGAGCGAATAGGA-3’ |
| Semi-quantitative RT-PCR | |
| β-galactosidase F | 5’-TTCACTGGCCGTCGTTTTACAACGTCGTGA-3’ |
| β-galactosidase R | 5’-ATGTGAGCGAGTAACAACCCGTCGGATTCT-3’ |
| Rp49 F | 5’-TACAGGCCCAAGATCGTGAA-3’ |
| Rp49 R | 5’-ACGTTGTGCACCAGGAACTT-3’ |
| dBCAS2 F | 5’-CGAAGTTATTGTGGATGCTCTGCCC-3’ |
| dBCAS2 R | 5’-CGGACACCTCCGACAATTTGCCCGAT-3’ |
